# Painless PDT using 10% aminolevulinate gel and red light: A pilot clinical trial of short-contact protocols to reduce discomfort during illumination

**DOI:** 10.1016/j.pdpdt.2025.104698

**Published:** 2025-06-26

**Authors:** Jessica S. Johnson, Jackson Hanna, Amy S. Nowacki, Sanjay Anand, Alan S. Shen, Edward V. Maytin

**Affiliations:** a Cleveland Clinic Lerner College of Medicine of Case Western Reserve University School of Medicine, Cleveland, OH, USA; b Department of Dermatology, OH, USA; c Department of Biomedical Engineering, OH, USA; d Department of Quantitative Health Sciences, Cleveland Clinic, Cleveland, OH, USA

**Keywords:** Photodynamic therapy, Aminolevulinic acid, Actinic keratoses, Red light, Pain

## Abstract

**Background:**

Traditional red light photodynamic therapy for actinic keratoses (AK) causes significant pain during illumination.

**Objective:**

To evaluate new short-incubation red light PDT regimens for their pain and efficacy characteristics.

**Methods:**

Patients (*n* = 30) with facial AK were treated with 10 % ALA gel (no occlusion) and red light (635 nm), after randomization as follows [incubation time, illumination time (fluence)]: Group A [10 min, 20 min (74 J/cm^2^)]; Group B [20 min, 10 min (37 J/cm^2^)]; Group C [1 hour, 10 min (37 J/cm^2^)]. Pain and AK lesion counts were recorded during two PDT treatments spaced 8 weeks apart. Final lesion counts were at 3–6 months.

**Results:**

At both treatment visits, Group A and B patients experienced negligible pain that was statistically less than that of Group C patients. AK clearance after two PDT treatments was robust and similar amongst the three treatment groups (76 % for Group A, 74 % for Group B, and 82 % for Group C). Using a non-inferiority margin of 15 %, lesion reduction in Group A was statistically non-inferior to Group C after one PDT treatment.

**Limitations:**

Efficacy rates of the new short incubation protocols (done without occlusion) were not compared directly with the long (3-hour) FDA-approved protocol that is done with occlusion.

**Conclusion:**

Short-contact red light PDT regimens are essentially painless and appear to provide very good AK lesion clearance.

**Clinical trial registration:**

NCT06027619 (date of trial registration: 31 August 2023).

## 1. Introduction

Squamous cell carcinoma (SCC) typically appears in sun-exposed field-cancerized skin, developing from pre-cancerous lesions called actinic keratoses (AK) 65 % – 97 % of the time. Conversely, anywhere from 0.5 – 20 % of AK lesions develop into SCC [1]. Therefore, treatment of AK lesions is critical to reduce progression to SCC. Photodynamic therapy (PDT) is a field treatment that utilizes a pro-drug, either aminolevulinic acid (5-ALA) or its methyl ester (m-ALA), in combination with visible light [2]. After topical application, ALA is selectively taken up by neoplastic cells and converted into protoporphyrin IX (PpIX), the actual photosensitizer. Illumination of PpIX with the appropriate wavelengths of visible light generates reactive oxygen species that damage mitochondrial membranes, leading to cell death [3]. Advantages of PDT over other AK treatment methods are its ability to target neoplastic cells in the entire treatment field, lack of scarring, and lack of mutagenic potential [4]. Downsides of the current Food and Drug Administration (FDA) approved PDT protocols include the long ALA incubation times (3 h for red light PDT or 14–18 h for blue light PDT) and the stinging pain that patients experience during illumination, as a result of the long incubation times [5]. Both problems hinder general acceptance of PDT by providers and patients and its overall implementation in dermatology offices.

In fact, pain is such a barrier to utilizing PDT that off-label usage of the technique, with shorter incubation times, is common practice and there is no standard for incubation/illumination that is universally utilized [6]. Fortunately, a number of recent studies show that long incubation times may not be necessary to achieve AK lesion clearance with PDT. A bilaterally controlled intra-patient study in 2020 compared a conventional regimen (c-PDT; 1 hour incubation with 20 % ALA) to a modified regimen (m-PDT) in which blue light was started immediately after ALA application and illumination continued for either 30, 45 or 60 min. Patients reported significantly less pain with m-PDT than with c-PDT, yet AK lesion clearance was similar across all treatment protocols [7]. The above-described blue light study was partially inspired by the results of “daylight PDT” studies in Europe, wherein AK patients were preincubated with 30 min of methyl-ALA and then exposed to outdoor sunlight; that short-incubation regime was shown to be non-inferior to conventional long-incubation (3 hour) red light PDT [8–10]. In a recent 2023 study by Ruiz et al., patients were illuminated for 10 min with traditional red light after a 30-minute incubation with 10 % ALA gel, and showed low pain scores and similar efficacy in AK lesion clearance when compared to daylight PDT (30 min ALA pre-incubation, followed by 2 hr of sunlight) [10]. Additional studies investigating m-PDT regimens with blue light have used a variety of incubation times between 0 – 15 min and also reported minimal pain in the short-incubation group [11–13]. During all of these short-incubation/simultaneous illumination protocols, pain may be absent because PpIX is being continuously destroyed due to photobleaching, thereby preventing PpIX from accumulating and diffusing into local nerves [14,15].

Red light is the standard of PDT care globally, while blue light (417 nm) is still the most commonly used light source for PDT in the United States. Red light (635 nm) has the advantage of penetrating deeper into lesions and is FDA-approved for PDT with the 10 % ALA gel formulation [16]. 10 % ALA gel (*ALA-gel*) is a nano-emulsion that provides increased stability of the active ingredient and has increased lipophilic properties, thereby enhancing ALA uptake into AK lesions [17–19]. In the FDA-approved protocol, ALA-gel is applied for 3 h under an occlusive dressing, followed by 10 min of red-light exposure. However, this protocol is associated with significant pain. Based on the prior findings for m-PDT, we designed a study with 3 arms to compare different timing regimens of 10 % ALA gel and red light. Two arms (Groups A and B) involved short ALA incubation times while the third (Group C) used a relatively longer ALA incubation but still not as long as the FDA-approved 3-hr protocol (for reasons explained below). The goal was to identify an optimal parameter combination that is effective, yet less painful than the current FDA-approved regimen.

## 2. Methods

### 2.1. Study design

A prospective, randomized clinical pilot trial was conducted at a single academic center. Patients were randomly assigned to one of 3 treatment arms (ALA-gel incubation time, red light illumination dose): Group A: 10 min, 20 min (74 J/cm^2^); Group B: 20 min, 10 min (37 J/cm^2^); Group C: 1 hour, 10 min (37 J/cm^2^). For our study, Group C served as a proxy for the long-incubation regimen. With no standard incubation/illumination scheme utilized in clinical practice, and the established literature showing effective AK lesion clearance with shortened protocols, we felt it unethical to inflict pain on our patients by utilizing the FDA approved 3-hour illumination scheme as the control. In each study group, two PDT treatments were administered 8 weeks apart. Participants recruited from our dermatology clinics gave informed consent before enrollment. The study was approved by the Cleveland Clinic’s Institutional Review Board (IRB #23–636) and registered with ClinicalTrials.gov (NCT06027619).

### 2.2. Study population

Patients with at least 10 AK lesions on the face were recruited between September 2023 and March 2024. Follow-ups occurred until July 2024. Exclusion criteria were age < 18 years, pregnant or nursing, concurrent use of other topical treatments for AK, history of photosensitivity, or hypersensitivity to ALA-gel.

### 2.3. Randomization

A block randomization scheme, supervised by the research pharmacy, was used to assign exactly 10 patients to each treatment group. Study clinicians were blind to the group assignment until just before treatment began.

### 2.4. Interventions

**Fig. 1.**
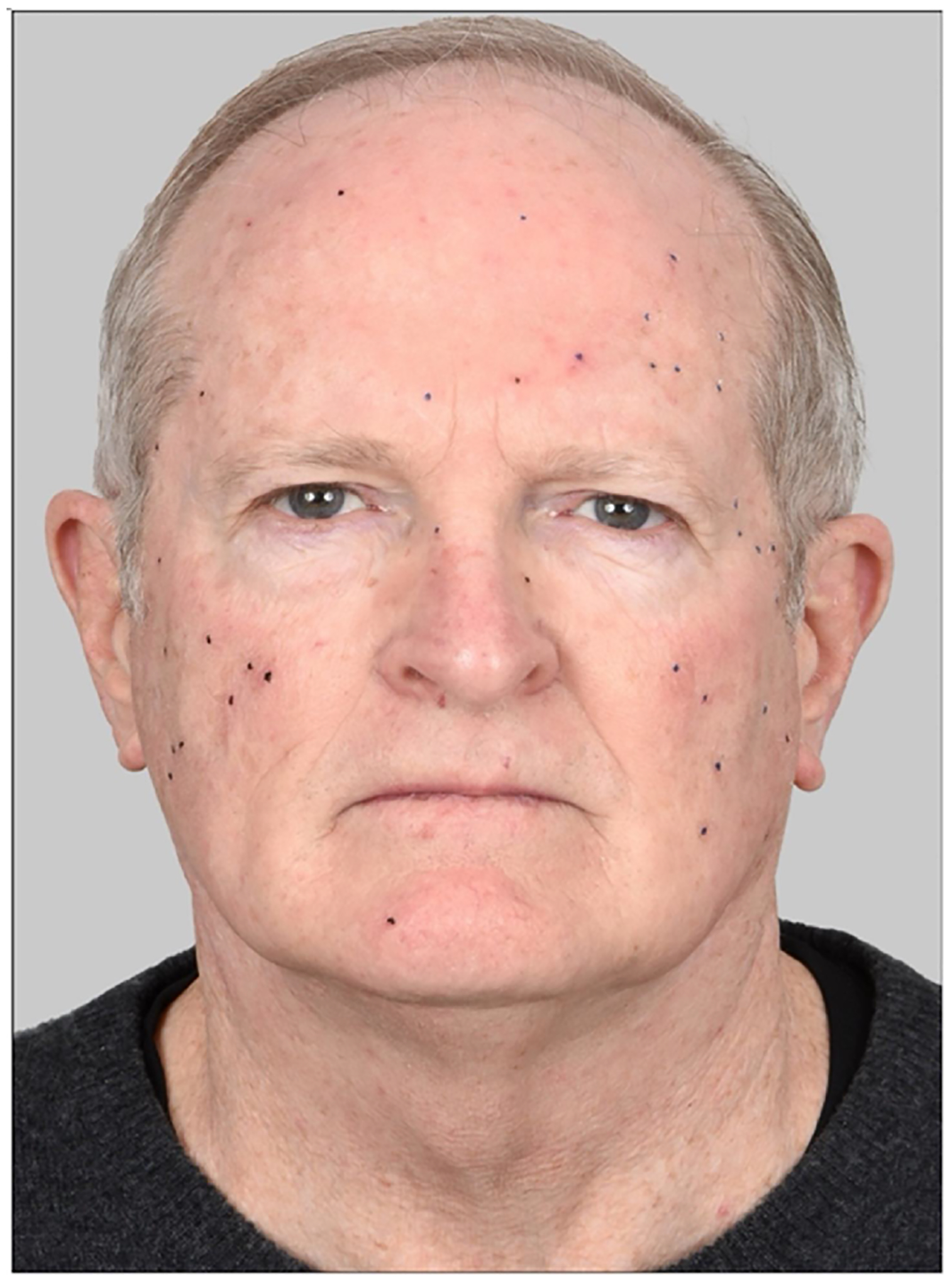
Photograph of a representative patient from Group A taken before PDT. Black dots are pen marks indicating the location of individual AK lesions.

FDA approved Aminolevulinic acid 10 % nanoemulsion (ALA gel, Ameluz^®^) and BF-RhodoLED^®^ red-light panels (Biofrontera Inc, 635 nm; irradiance ~60 mW/cm^2^) were utilized. There were four study visits. At Visit 1 (Day 0), facial AK lesions were counted, marked with a pen, and documented by a professional photographer (Fig. 1). Sandpaper curettage was performed on all AK lesions with palpable scale [20]; the majority of our patients’ lesions were Olsen grade 1 or 2, with fewer than 10 % being grade 3. PDT treatment was then administered using two BF-RhodoLED^®^ lamps. The surface area covered by a single lamp was relatively small (i.e., less than a third of the face), so to illuminate the entire face it was necessary to develop a reproducible method of providing uniform coverage of a curved surface, including forehead/temples (upper face) and cheeks/nose (lower face). To accomplish this, we used two lamps simultaneously, with one lamp positioned on either side of the patient. The first lamp’s LED panel (mounted on its base and movable arm) was initially placed at forehead/temple level on the left, then moved horizontally to the middle of the forehead after 33 % of exposure time; and finally moved to the right side after 66 % of exposure had elapsed. This provided coverage of the entire upper face. The second lamp was similarly moved so as to illuminate the lower half of the face (cheeks/nose), but moving in the opposite direction to prevent the two mounted lamps from colliding with each other. At Visit 2 (Day 3 ± 1 day), patients were re-photographed to document inflammation post PDT. At Visit 3 (Week 8 ± 1 week), lesions were counted/photographed again, and PDT performed again exactly as before. Final lesion counts were done at Visit 4 (3 – 6 months). AK lesions were identified using clinical criteria (scale, erythema, roughness to palpation), simultaneously by two investigators (JJ and EM), and average counts reported.

Patients reported their pain levels during PDT illumination at 1 min, 5 min, and end of treatment. The cooling fan in the BF-RhodoLED^®^ unit was run at the highest setting for all patients. Patients were sent home wearing a wide-brim hat, instructions to avoid sunlight for 48 h, and a side effects questionnaire to complete.

### 2.5. Outcome measures

Our study had two primary endpoints. Pain during illumination was measured on an eleven-point scale (0 = no pain, 10 = intolerable) and the maximum value at each visit (V1 and V3) was utilized for final analysis. AK lesion reduction was defined as the percent reduction in AK counts at Visit 3 and Visit 4, relative to counts at Visit 1 (baseline).

**Fig. 2.**
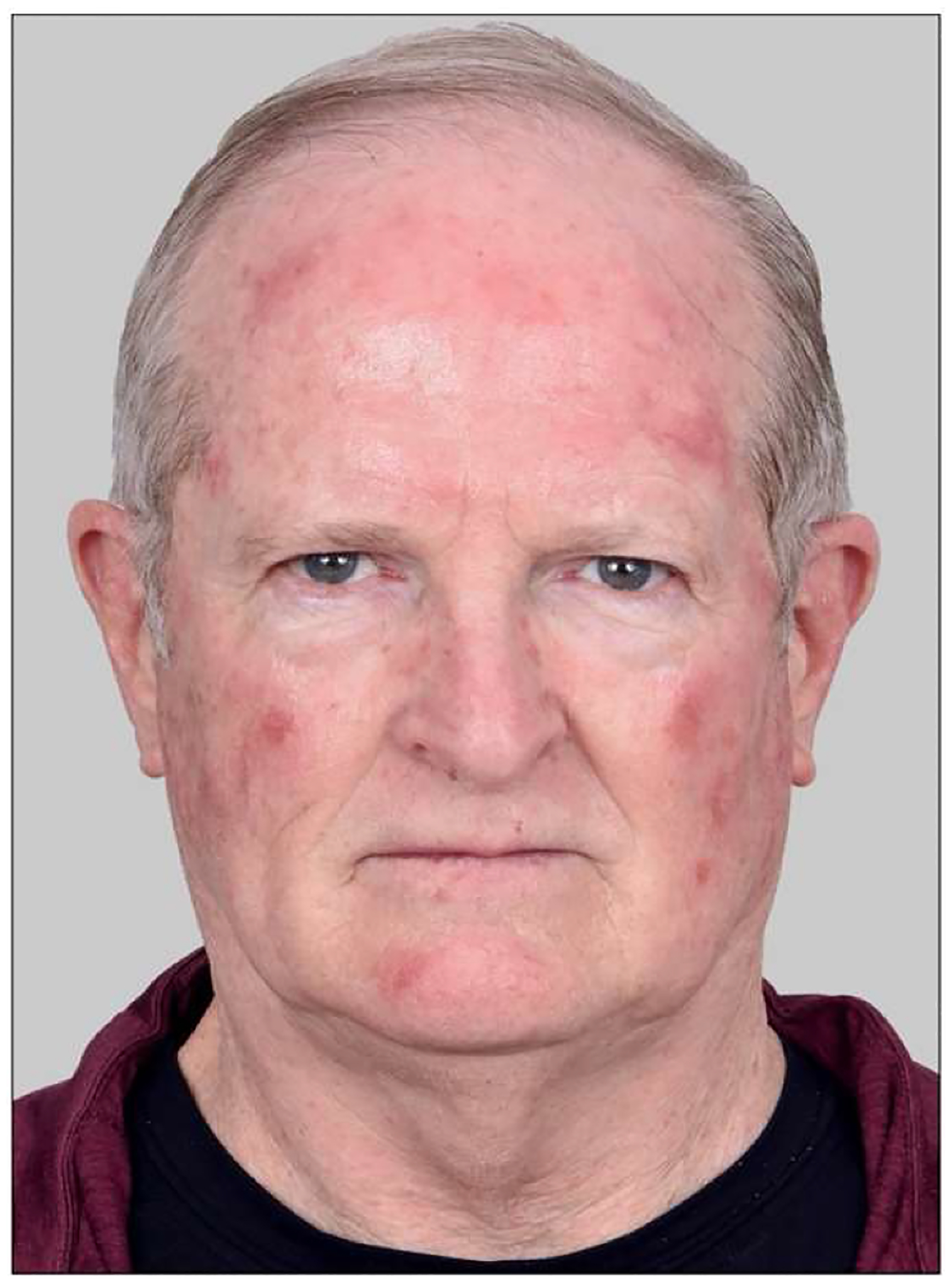
Photograph of the same patient at 3 days post-PDT, demonstrating his erythema response. The mean change in erythema scores for this patient were: forehead (4.25), temples (3.75), cheeks (4), supralabial region (0.5), nose (1.75).

Secondary endpoints were erythema (redness, measured from photographs) and other inflammatory side effects (determined by patients in a self-reported questionnaire). Erythema was determined from photographs taken at baseline (Visit 1) and at three days post-PDT (Visit 2), as judged by four independent reviewers. Utilizing a previously published erythema scale [21], the reviewers scored each of five anatomic areas on the face, at Visit 1 (pre-PDT) and Visit 2 (post-PDT). The difference between these scores was reported as an erythema increase. An example of this semiquantitative photographic evaluation of erythema is shown in Figs. 1 and 2. Other side effects were recorded by the patients for 6 days in their take-home questionnaire.

### 2.6. Statistical analysis

During the first PDT treatment (Visit 1) patients would be expected to have higher AK lesion counts than at the second PDT treatment (Visit 3) and because pain levels tend to correlate with neoplastic burden [22], we expect the pain ratings between Visit 1 and Visit 3 to be different and distinct. Thus, to compare maximum pain levels across the three groups, a one-way ANOVA was utilized at Visit 1 and Visit 3 separately.

For AK lesion clearance, non-inferiority between treatment groups was tested using a confidence interval (CI) approach applied to a linear mixed-effect model, accommodating the repeated measures within patient. Non-inferiority of the short-contact treatment groups (Group A and Group B) to long-contact therapy (Group C), was claimed if the lower bound of the two-sided 90 % CI [for the percent difference in mean AK clearance] (equivalent to the lower bound of a one-sided 95 % CI) was greater than negative 15 %, the non-inferiority margin. The non-inferiority margin was selected based on prior literature and clinical expertise [7]. Acknowledging that the number of AK lesions varies substantially amongst patients, with a minimum of 10 AK lesions needed for enrollment in this study, this 15 % absolute difference equates to ~1–2 AK lesions for the minimum in our study. An interaction term was incorporated to evaluate whether the treatment group effect on AK lesion clearance was modified by visit number (Visit 3, Visit 4). If evidence of a statistically significant interaction was present, the treatment group effects were estimated at each visit separately.

Side effects were summarized using descriptive statistics. Erythema levels were compared amongst treatment groups via ANOVA. If values were significant, Tukey’s test was utilized for inter-group pairwise comparisons.

A significance level of 5 % was utilized. This trial was a pilot study and thus we did not adjust for multiple testing with two co-primary endpoints, pain and AK lesion clearance. Analyses were conducted with SAS version 9.4 statistical software (SAS Institute, Cary, NC).

### 2.7. Sample size justification

The study was powered to evaluate the co-primary endpoint of AK lesion clearance, using estimates of the effect size and variability from prior studies to test for non-inferiority between each of the shortened incubation PDT regimens (Group A, or Group B), and the long incubation PDT regimen (Group C). We based estimates for our long incubation regimen, on data reported for the original red light 3-hour incubation clinical trial, a mean AK lesion clearance rate of 78 % [17]. To estimate the clinical response one might see in a pilot study, we extrapolated results from our earlier study of short ALA-incubation and blue light [7]. That prior study reported an AK lesion clearance rate of 61 %. A sample size calculation for a non-inferiority test with a margin of −15 %, mean clearance rate of 78 % in the long incubation group, mean clearance rate of 61 % in the short incubation group, a standard deviation of 30 %, power of 80 %, and an alpha of 0.05, resulting in 10 subjects per group (30 subjects total) [7].

A sample size calculation was additionally completed for the co-primary endpoint, pain, with a one-way ANOVA overall F test, Bonferroni adjusted for the two timepoints (Visit 1, Visit 3) and assuming a 10 % drop-out rate. Utilizing estimates for the effect size and variability from prior studies [7]: mean of 3.57 in the long incubation group, mean of 0.52 in the short incubation group, an overall standard deviation of 0.6, power of 80 %, and an alpha of 0.05, resulted in a sample size of 3 per group (9 subjects total). The higher sample size of 30 was thus utilized.

## 3. Results

Thirty patients were recruited. Of these, one dropped out (from Group C, due to pain). All raw data from the clinical trial are shown in Table 1. Demographics were similar amongst the 3 treatment groups; participants were largely male (86 %), older (mean age of 70.1), and had received prior treatment for AK lesions (Table 2). The patients had, on average, 46 AK lesions (range, 15–99).

**Fig. 3.**
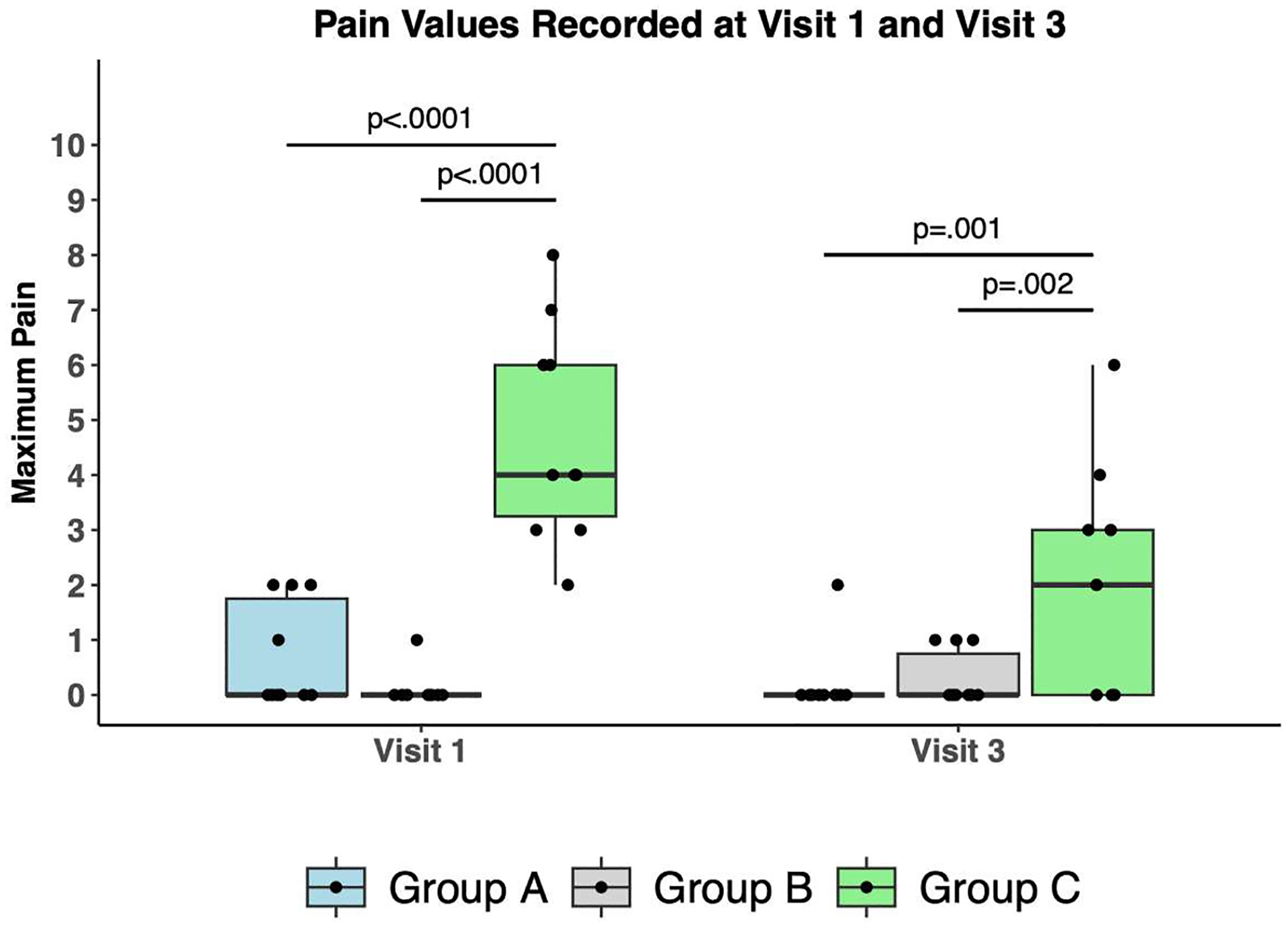
Maximum pain values (mean ± standard error) were recorded at each PDT session and averaged within each treatment cohort. (Patient who dropped out was excluded from analysis). *Brackets:* Post-hoc multiple comparison tests; p-values are indicated.

Evaluation of maximum pain during illumination at Visit 1 and Visit 3 (Fig. 3) showed that Group A and Group B patients had significantly lower levels of pain compared to Group C, at Visit 1 (mean maximum pain 0.7, 0.1, 4.3 for Groups A, B, C, respectively; *P* < .001) and also at Visit 3 (mean maximum pain 0.2, 0.3, 2.2 for Groups A, B, C, respectively; *P* = .002).

**Fig. 4.**
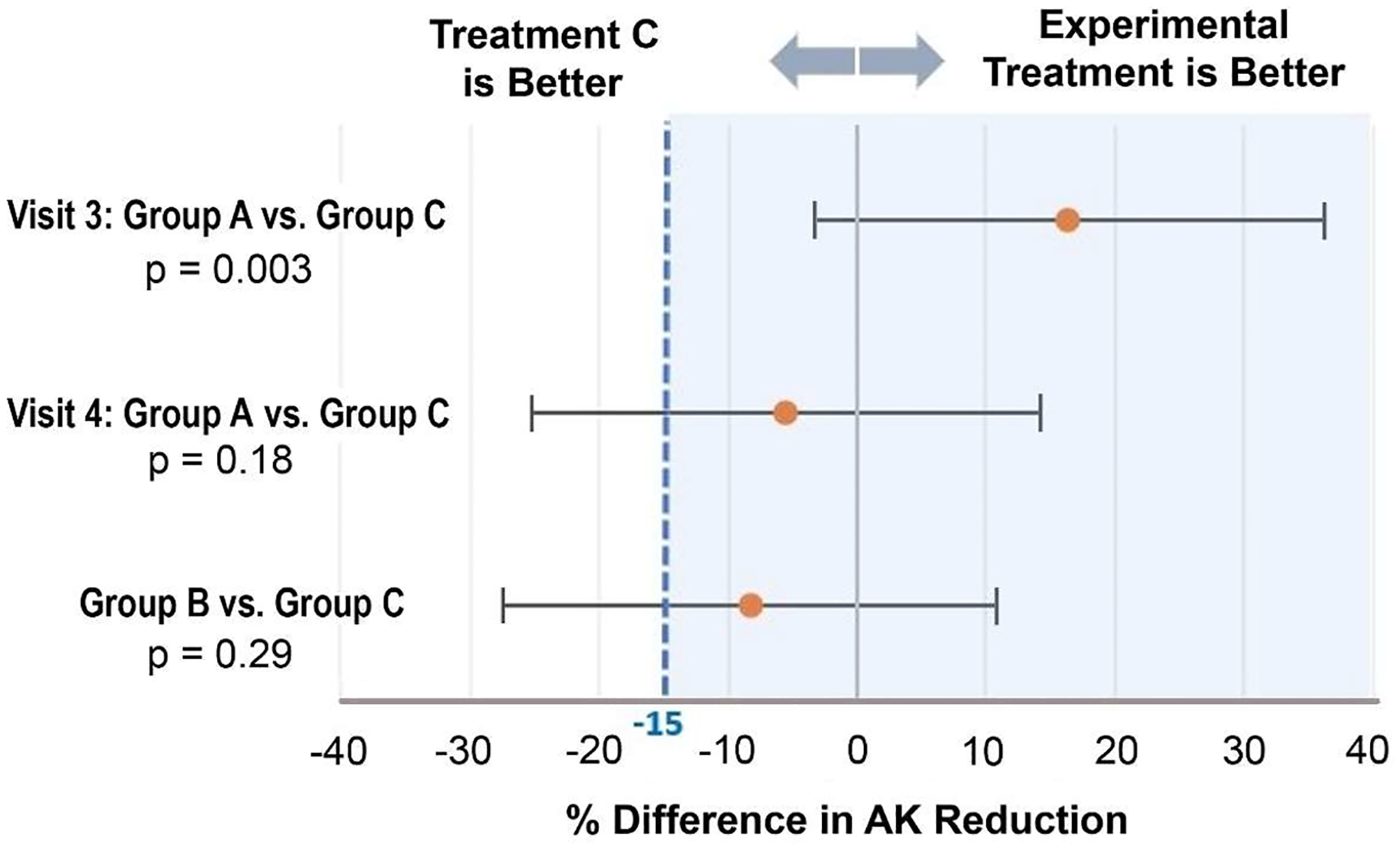
Forest plot demonstrating the comparison of reduction in AK lesions between different treatment groups at a clinical efficacy margin of −15 % and a 90 % CI interval.

AK lesions were counted and the relative lesion reduction calculated after one (Visit 3) or two (Visit 4) PDT treatments. The short-incubation groups, A and B, were evaluated for non-inferiority to Group C in terms of AK lesion clearance. In the A vs. C comparison, the interaction term for the relationship between treatment group (A, C) and visit number (3, 4) was significant (*P* = .046), and thus in the A vs. C comparison a treatment group effect was estimated for each visit. In contrast, in the B vs. C comparison, the interaction term for the relationship between treatment group (B, C) and visit number (3, 4) was not statistically significant (*P* = .96), and thus the treatment group effect was estimated overall (averaged across visits). Lesion clearance for Groups A, B, and C after one PDT treatment were 59 %, 34 %, and 43 % respectively, and after two PDT treatments were 76 %, 74 %, and 82 % respectively. Statistical testing for non-inferiority was affirmative for the comparison of Groups A and C after one PDT treatment, but not when comparing the various groups after two PDT treatments (Fig. 4). Notably, as this was a pilot study with limited sample size, the majority of the confidence intervals lay in the noninferiority region and may with more precision (larger sample size) support practical utility.

Side effects were recorded for 6 days following PDT treatment (Table 3). Of the 28/29 (97 %) participants who returned their side effects journal, 100 % reported at least 1 side effect. Redness, itching, and burning were listed most frequently.

The difference in erythema, measured pre- and post-PDT, was highest in Group C overall and was significantly higher (all *P* < .05) than Group B for forehead, temples, and cheeks and Group A for temples and cheeks (Table 4). Group A was significantly higher than Group B for temples and cheeks (Table 4).

## 4. Discussion

We report on three modified PDT (m-PDT) regimens using red light and 10 % ALA gel for the treatment of AK lesions. Of the 30 patients enrolled, only one patient dropped out. That patient was assigned to Group C and found the treatment too painful to receive the second PDT treatment. Her experience highlights the importance of our results, namely that the short-incubation protocols (Groups A and B) resulted in significantly less pain as compared to the long-incubation protocol (Group C), a finding that generally agrees with prior blue light m-PDT studies [7,11–13] and aligns with other recently described red light PDT protocols that are practically painless. A study by Ruiz et al. in which patients were irradiated for 10 min using red-light (635 nm, 37 J/cm^2^) after a 30-minute incubation with 10 % ALA gel had lesion clearance of 57 % ± 25 % at 6 months after one PDT treatment and a maximum pain score of 1.8 out of 10 [10]. In a study by Mordon et al. [23], a 30 min methyl-ALA incubation was followed by red light illumination using a fabric-based biophotonic device (total light dose of 12 J/cm^2^ delivered over a 2.5 hour period); the average pain score was 0.3 and after two PDT sessions given 3 months apart; AK lesion clearance rates were 79.3 % at 3 months and 94.2 % at 6 months. Li et al. used a 30 min incubation of 10 % ALA followed by 120 min of red light (total dose 288 J/cm^2^). At each of three PDT treatments (given 2 weeks apart), pain scores were 0 – 2, and the clearance rate was 91.6 % at 1 month [24].

Our results compare favorably with the foundational studies of 10 % ALA-gel and red light, which employed a 3-hour incubation with occlusion, and analyzed lesions only within a limited target area of ~20 cm^2^ area [17,18,25]. After two treatments using a narrow-emission spectrum (635 nm) for 10 min (37 J/cm^2^), lesion clearance rates surpassed 90 % [18]. However, 96 % of participants reported pain, with mean pain levels of 5.5/10 [18]. In contrast, our study was designed to simulate a real-world clinical setting in which PDT is applied to the entire face rather than to a limited target area. Furthermore, we utilized no occlusive dressings. Clearance rates after two PDT treatments (76 % for Group A, 74 % for Group B, 82 % for Group C) were lower in our study than in the earlier reports [17,18,25], but this can be easily explained by the differing treatment methods. These newly described red light m-PDT regimens (Group A in particular) should be much easier for patients and more convenient for clinicians, while still providing outstanding results.

In our study, erythema was monitored as a marker of the PDT-induced inflammatory response. Patients in Group C had the most erythema overall, Group A had somewhat less, and Group B the least. Based upon recent studies in a murine AK model showing that m-PDT works primarily through immune cell recruitment rather than by generation of reactive oxygen species and apoptosis [26,27], together with a clinical study showing that post-PDT erythema significantly correlates with areas of highest AK lesion burden [21], we conclude that the robust erythema observed in Groups A and C provides strong evidence that red light m-PDT is generating an immune response against AK lesions.

The ability of our study to demonstrate statistical non-inferiority, in terms of AK lesion reduction, was limited by a small sample size. For our pilot non inferiority study, AK lesion reduction was appropriate but another outcome measure for AK would be the proportion of patients with complete lesion clearance. The latter endpoint as well as longer follow-up times will be considered for future phase I/II studies. Despite limitations, the current results have important clinical implications. Short-incubation regimens with red light PDT can be nearly painless, and substantially reduce office wait times while still achieving acceptable AK clearance rates. Of note, Group A received double the illumination time (20 min) and showed better efficacy than Group B (10 min), suggesting that energy delivery can play an important role in AK clearance. Even longer illumination times, from 30 – 60 min, were used in previous m-PDT studies using blue light [7,13]. Because red light has a ~10-fold lower absorption coefficient than blue light [28], we speculate that illumination with red light can be safely lengthened to maintain a painless treatment despite the relatively higher energy delivery. When comparing incubation times and lesion clearance, Group C in our study (60 min ALA incubation) showed the highest AK clearance (84 %), but this was also associated with the highest pain scores (average of 4.3 at V1 and 2.2 at V3). Note that this is still less than maximum pain levels in studies utilizing the 3 h-incubation (current protocol approved by the FDA; average pain score of 5.5) which provided an average AK lesion clearance rate of 78 % (± 15 %). Therefore, the Group C (1 hour ALA) protocol may be acceptable to some patients/providers to achieve increased efficacy, especially if the patients have moderate or severe AK lesions.

To optimize the balance between efficacy and tolerability in red light PDT protocols, we believe that future research should focus on short incubations and long illumination times as the best paradigm. It would also be interesting to see whether this approach yields similar results for AKs found on the scalp and extremities. In summary, short incubation red light PDT holds promise as a convenient, effective, and tolerable treatment for patients with extensive field cancerization of the skin.

## Figures and Tables

**Table 1:** Complete dataset for all patients enrolled in study, ClinicalTrials.gov NCT06027619.

Record ID	Sex	Age (Years)	History of Skin Cancer?	Prior treatment for AKs?	Study Group	AK Lesion Counts			Max Pain Level	
						Visit 1	Visit 3	Visit 4	Visit 1	Visit 3
1	Male	72	Yes	Yes	B	41	21	13	0	1
2	Male	66	Yes	Yes	C	57	22	15	4	4
3	Male	63	No	Yes	B	15	5	3	0	0
4	Male	68	Yes	Yes	A	84	30	19	2	0
5	Male	64	Yes	Yes	A	46	29	21	2	2
6	Male	68	Yes	Yes	C	64	69	27	3	2
7	Male	84	Yes	Yes	B	77	72	20	0	0
8	Male	74	No	Yes	C	16	19	5	3	0
9	Male	66	Yes	Yes	C	57	32	8	2	0
10	Male	65	No	Yes	A	67	32	19	0	0
11	Male	64	Yes	Yes	A	38	16	4	1	0
12	Male	70	Yes	Yes	B	26	23	9	0	1
13	Male	79	Yes	Yes	C	27	23	7	4	2
14	Male	61	Yes	Yes	B	48	35	8	1	0
15	Male	66	Yes	Yes	B	42	12	2	0	0
16	Male	56	Yes	Yes	A	15	6	1	0	0
17	Female	76	Yes	Yes	C	29	Patient dropout		8	–
18	Male	72	Yes	Yes	A	52	18	32	0	0
19	Male	74	No	Yes	C	44	13	3	6	0
20	Male	79	Yes	Yes	A	99	31	23	2	0
21	Male	74	Yes	Yes	B	40	12	5	0	0
22	Female	69	Yes	Yes	C	45	11	2	4	3
23	Male	73	Yes	Yes	A	38	15	3	0	0
24	Male	64	No	Yes	B	28	38	10	0	0
25	Male	76	Yes	Yes	B	23	6	2	0	1
26	Male	78	Yes	Yes	C	68	15	5	7	6
27	Male	69	Yes	Yes	B	31	16	12	0	0
28	Female	62	No	Yes	C	56	19	2	6	3
29	Male	72	Yes	Yes	A	47	14	7	0	0
30	Female	80	Yes	Yes	A	62	23	5	0	0

**Table 2:** Demographic information for enrolled patients.

Group assignment —	A (*n* = 10)	B (*n* = 10)	C (*n* = 10)*
Age (Mean, SD)	69.3 (7.4)	69.9 (7.0)	71.2 (5.8)
Male	9 (90 %)	10 (100 %)	7 (70 %)
History of skin cancer?	9 (90 %)	8 (80 %)	7 (70 %)
AK lesions (Mean, SD)	54.8 (24.3)	37.1 (17.3)	46.3 (17.3)
AKs previously treated?	10 (100 %)	10 (100 %)	10 (100 %)

* Group C includes information from the patient who dropped out after one PDT treatment.

**Table 3:** Percent of study patients experiencing side effects at 2 days after PDT.

	Group A (*n* = 9)*	Group B (*n* = 10)*	Group C (*n* = 9)*
Redness	100 %	90 %	100 %
Pain	22 %	0 %	33 %
Burning	56 %	10 %	33 %
Itching	44 %	50 %	56 %
Stinging	44 %	10 %	33 %
Swelling	0 %	0 %	33 %
Crusting	11 %	10 %	11 %
Peeling	11 %	10 %	22 %
Blisters	0 %	0 %	22 %
Bleeding	0 %	10 %	11 %
Ulcers	0 %	0 %	11 %
Other	0 %	10 %	22 %

* n represents the number of surveys that were returned.

**Table 4:** Average increase in erythema score at 3 days post-PDT, by treatment group*.

Anatomic Location	Mean Erythema Increase, Mean (SE)			ANOVA p-value**	Tukey’s Post-hoc Comparisons***
	Group A	Group B	Group C		
Forehead	1.95 (0.39)	1.03 (0.20)	2.80 (0.29)	0.0013	B vs C
Temples	2.00 (0.36)	0.65 (0.25)	2.53 (0.38)	0.0017	B vs C; B vs A
Cheeks	1.90 (0.35)	1.25 (0.19)	3.00 (0.26)	0.0005	B vs. C; A vs C
Supralabial	0.38 (0.19)	0.11 (0.08)	1.25 (0.44)	0.14	–
Nose	1.23 (0.27)	0.58 (0.22)	1.45 (0.31)	0.17	–

* Erythema rated by four independent evaluators of high-resolution photographs taken of each patient before and after PDT, was determined using a 6-point scale (normal skin = 0, mild erythema = 1 or 2, moderate erythema 3 or 4, intense erythema = 5 or 6). For each patient, a photograph at baseline (pre-PDT) and another photograph taken 3 days after PDT (post-PDT) were evaluated, and the difference between the two scores was defined as the average increase in erythema due to PDT. The erythema increase values, scored by each evaluator, were averaged and reported in the table as the mean ± standard error of the mean.

** Tukey’s test was utilized if p-value for group comparison was <0.05.

*** Comparisons significant at the 0.05 level.
